# A case of contralateral inguinal lymph node metastases from breast cancer

**DOI:** 10.1186/s40792-021-01181-z

**Published:** 2021-04-20

**Authors:** Takeo Kimoto, Norio Kohno, Akiko Okamoto, Kyosuke Ota, Takafumi Tani, Takeshi Kondo, Mari Nishio

**Affiliations:** 1 Department of Surgery, Kobe Kaisei Hospital, 3-11-15 Nada-ku-Shinoharakita-machi, Kobe, Hyogo 657-0068 Japan; 2 Department of Breast Surgery, Saiseikai Nakatsu Hospital, 2-10-39 Kita-ku-Shibata, Osaka, 530-0012 Japan; 3 Department of Breast Surgery, Kohnan Medical Center, 1-5-16 Higashinada-ku-Kamokogahara, Kobe, Hyogo 658-0064 Japan; 4 Division of Forensic Pathology, Department of Forensic Medicine, Kobe University Graduate School of Medicine, 7-5-2 chuo-ku-Kusunokicho, Kobe, Hyogo 650-0017 Japan; 5 Division of Pathology, Department of Pathology, Kobe University Graduate School of Medicine, 7-5-2 chuo-ku-Kusunokicho, Kobe, Hyogo 650-0017 Japan

**Keywords:** Inguinal lymph node metastasis, Breast cancer, Contralateral metastasis, Lymphatic pathway

## Abstract

**Background:**

Breast cancer is well known to tends to invade through the lymphatic chains mainly to the axillary and subclavian nodes or occasionally to the internal mammary nodes. However, inguinal lymph node metastasis from breast cancer is extremely rare.

**Case presentation:**

We have experienced a case of an 82-year-old woman showing left inguinal lymph node metastases from right breast cancer. Previously, she had received five times abdominal operations and left artificial bone head replacement for metamorphous hip-joint disease. Although the metastases were firstly detected 46 months after the breast surgery, they had already existed at the time of the breast operation, which was retrospectively re-evaluated by CT examination. The progression pattern of inguinal lymph node metastases had much correlated with that of the breast cancer. She underwent inguinal lymph node dissections. Pathological findings revealed them being compatible with breast cancer origin.

**Conclusions:**

This is the sixth case having been reported in English literature. Besides, this is the first case showing the contralateral spread to the primary breast cancer. One of the causes of this complex metastatic pattern is thought be ascribed to the previously performed prolific abdominal operations.

## Background

Breast cancer is well known to metastasize to the entire organs by hematological spread to such as the bone, lung, liver, and the brain and so on. It also tends to invade through the lymphatic chains mainly to the axillary nodes or occasionally to the internal mammary nodes. These lymphatic pathways have been reported to be altered after the treatments such as axillary lymph node dissection (ALND) and/or radiation (RT) [1–4]. The study of preoperative lymphoscintigraphies for the patients with ipsilateral breast cancer recurrences showed that aberrant lymphatic drainages were relatively common in patients with ALND and/or RT [2]. Indeed, there have been several reports showing the contralateral axillary or intramammary lymph node metastases [1, 2, 5]. However, the present case as metastasizing to the contralateral inguinal lymph node is far rarer. Because, in this case, the metastasis was relevant to neither breast surgery nor radiation, the previously performed prolific surgeries could influence the alterations of lymphatic pathways.

## Case presentation

**Fig. 1:**
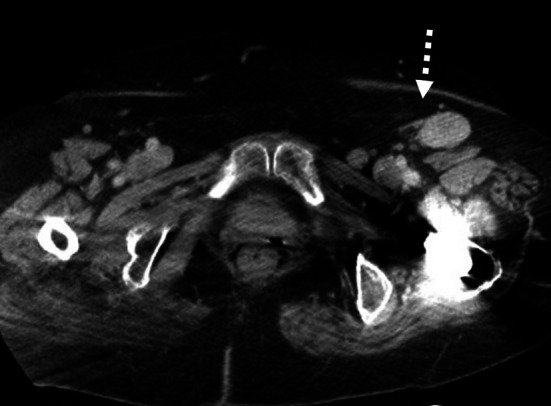
The increasing size and numbers of left inguinal lymph nodes were clearly presented in enhanced pelvic CT. The finding was first detected 46 months after the breast cancer operation

An 82-year-old woman was diagnosed as showing the increases of both size and numbers in left inguinal lymph nodes, among which the main one was 37 × 21 mm in size by CT examination (Fig. 1).

She had undergone the surgeries for transverse colon cancer (pathological T3N0M0: Stage II) and gastric cancer (pT1aN0M0: Stage IA) concomitantly performed by laparotomy 7 years before, and she had also received caesarian sections two times in her 20 s, hysterectomy for myoma in her 30 s, and mesh repair of incisional hernia by laparotomy 4 years before. She has also suffered from congenital right hip dislocation and had received artificial bone head replacement for left metamorphous hip-joint disease. Additionally, she had the operation for right breast cancer 46 months before, which was incidentally detected by CT for checking-up after the colon cancer operation. She received modified radical mastectomy. The intraoperative axillary sentinel lymph node (SLN) was positive for malignancy, so further dissections up to the low-axilla (lateral area from the border of pectoralis minor muscle) was added. The final pathology showed an invasive ductal cancer, 35 × 33 × 20 mm in size, predominant scirrhous type, and nuclear grade 1, and that seven out 23 axillary lymph nodes dissected were metastatic. The TNM Pathological Classification in UICC criterion was T2N2aM0, Stage IIIA. The immunohistochemical examination clarified it estrogen receptor (ER)-positive (89.4%), progesterone receptor (PgR)-positive (92.8%), and human epidermal growth factor receptor 2 (HER2)-negative (0), and Ki-67 labeling index being 16.1%. On 53 days after the operation, the persistent and intractable axillary lymphorrhea continuing even after her discharge made us to perform the reoperation, which was completed by suturing the opening of the axillary lymphatic duct. Thereafter, the lymphatic discharge was promptly ceased. Indeed, she had been long time suffering from diabetes mellitus and her activities of daily life (ADL) was extremely poor due to disproportionate obesity (141.5 cm in height, 93 kg in weight, BIM: 46.4). Then, she was considered being intolerant to the ordinal anthracycline-based adjuvant chemotherapy. Four cycles of gemcitabine were opted for adjuvant chemotherapy. Although the currently available data do not support the acceptance of gemcitabine as an adjuvant therapeutic option, gemcitabine has already demonstrated clinical efficacy in advanced or metastatic breast cancer with minimal toxicity even in elderly patients [6]. Additional radiotherapy to the axilla was also abandoned for the same reasons. The administration of aromatase inhibitor had been continuously challenged postoperatively.

**Fig. 2:**
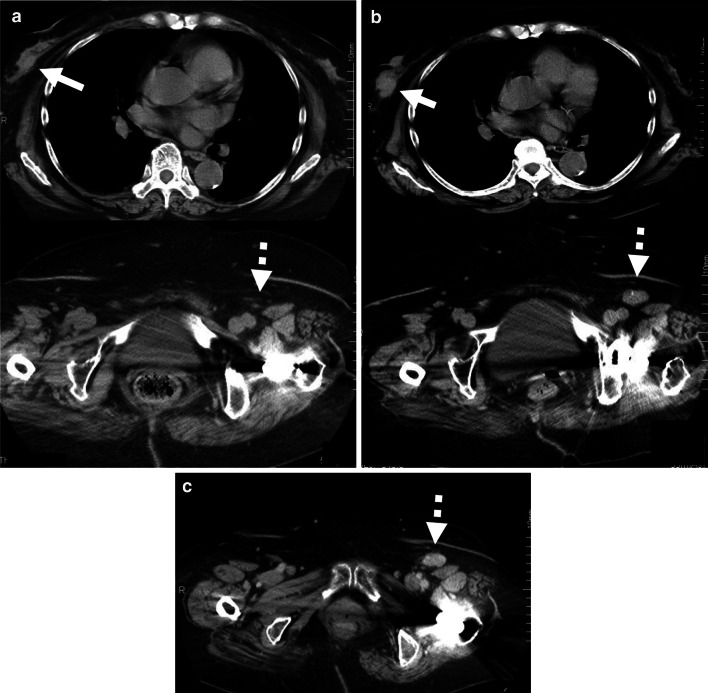
**a** The chest (above) and pelvic (below) CTs in 28 months before the breast cancer operation: right breast tumor can be barely identified (arrow), whereas left inguinal node swelling cannot be detected (dotted arrow). **b** The chest (above) and pelvic (below) CTs at the time of breast cancer operation: right breast tumor was getting to be apparent (arrow) in concordance with the left inguinal node swelling, 28 × 17 mm in size (dotted arrow) which had been overlooked. **c** The pelvic CT in 3 years after the breast cancer operation. As compared with the inguinal swelling shown in **b** (at the time of breast cancer surgery), the size of the nodes had still been dormant (dotted arrow). During these periods, adjuvant hormone therapy had been done in parallel

After the detection of inguinal lymph nodes swellings for the first time, we retrospectively scrutinized the series of CTs having been previously performed, which revealed that the unusual inguinal lymph node enlargement had already existed at the time of breast cancer operation. Intriguingly, the growing pattern of the nodes was very well correlated with that of the courses of breast cancer. At 28 months before the breast cancer operation, the checked-up CTs for colon cancer showed the slight enlarging breast tumor and the inguinal nodes being normal (Fig. 2a). Whereas at the detection of the apparently developing right breast cancer, the left inguinal node swelling was getting to grow up, which was overlooked (Fig. 2b). Interestingly, the inguinal node sizes seemed to have been dormant for at least 3 years after the breast operation, during which time adjuvant hormone therapy had been applied (Fig. 2c).

**Fig. 3:**
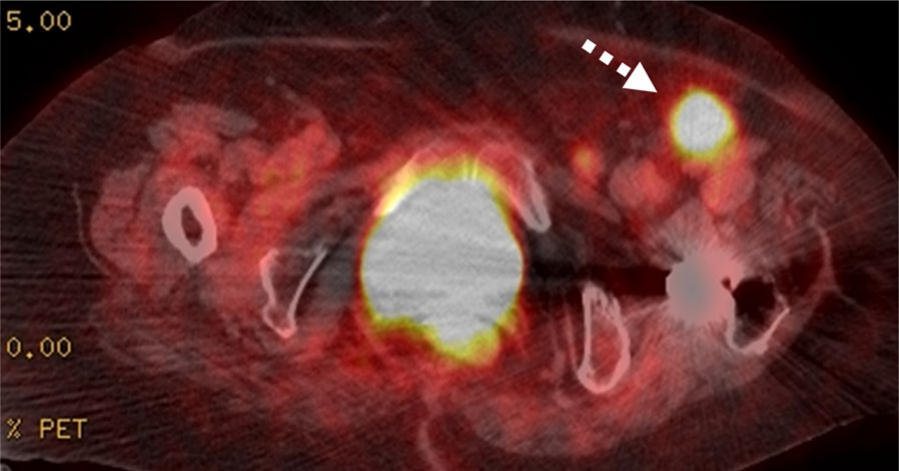
FDG accumulation was found only in the left inguinal region by PET (dotted arrow)

**Fig. 4:**
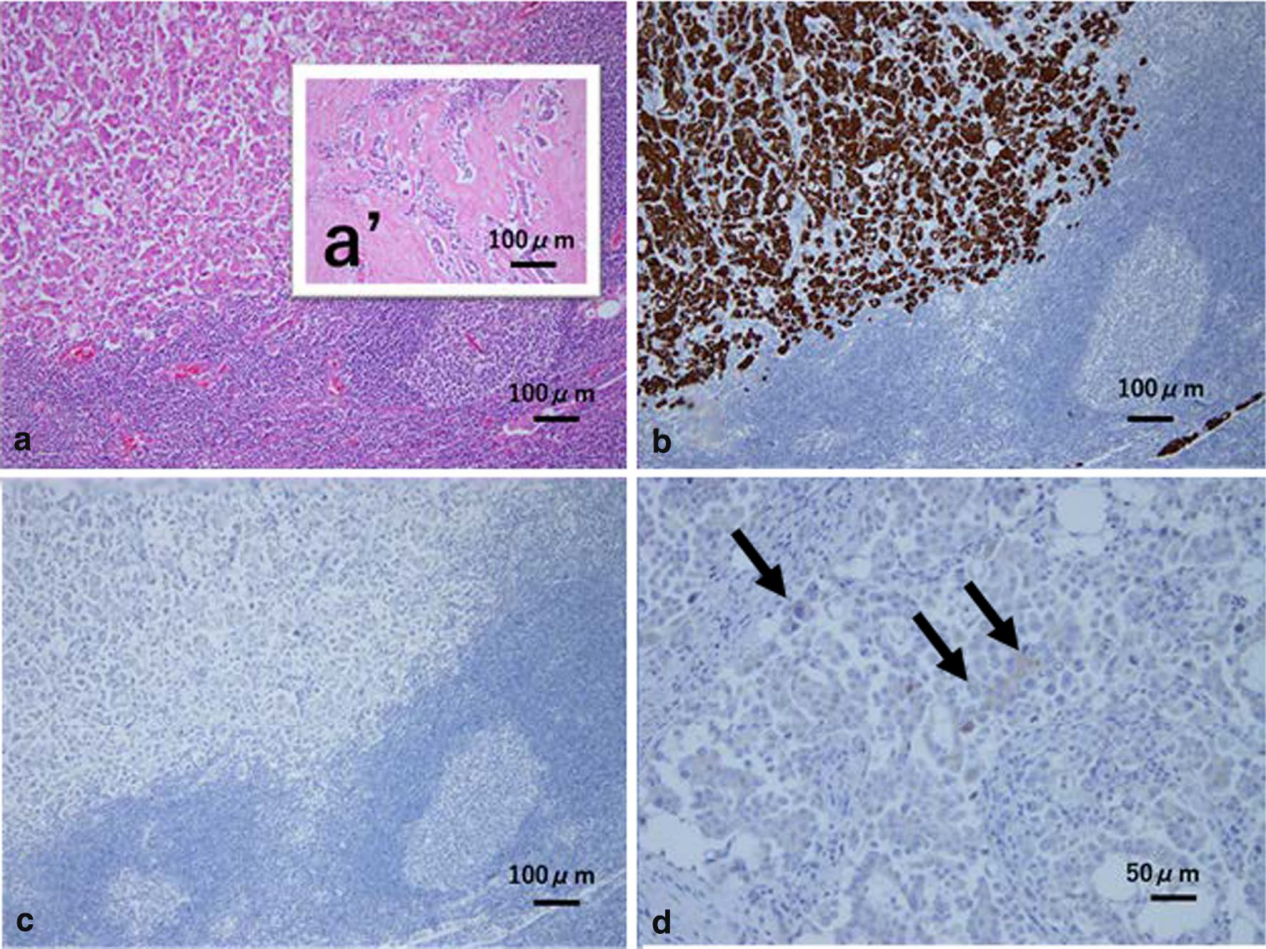
**a** Cancer cells forming micropapillary or compact nested foci were found to invade into the lymph node, which is compatible with primary breast cancer (hematoxylin and eosin (HE) × 100). **a’** Primary breast cancer showed predominant in scirrhous type. (HE × 100). **b** Cancer cells were CK 7-positive (× 100). **c** Cancer cells were CK 20-negative (× 100). **d** Mammaglobin-positive cells (arrows) (× 200)

After assuring the inguinal lymph node swellings, the core needle biopsy achieved into the main node showed it plausible for metastasis from the breast cancer. The PET–-CT revealed no other FDG accumulations than that in the left inguinal region (Fig. 3). Neither gynecological nor gastrointestinal examinations revealed abnormalities. There was no abnormal finding in the vulva. Then, superficial left inguinal lymph node dissections were performed with curative intention. The final histological result revealed that 13 out of 19 inguinal nodes were metastatic. Immunohistochemical profiles were cytokeratin (CK) 7-positive, CK20-negative, ER-positive (62.5%), PgR-negative, HER-2-negative (1 +), Ki-67 (29.9%), and human mammaglobin was slightly positive (Fig. 4). These findings indicated that the inguinal lymph node metastases were compatible with breast cancer origin [7–9]. To date, she has been receiving anti-estrogen drug (fulvestrant) as adjuvant.

## Discussion

Inguinal lymph node metastasis from breast cancer is extremely rare [10–13]. Immunohistochemistry demonstrated the positive expression of CK7 and ER, and the absence of CK20 in our case. These expressions indicate the possibilities of gastrointestinal and urinary origins to be substantially excluded from the inguinal metastases. On the contrary, most of the breast cancers show these expression patterns [7, 8]. Additionally, mammaglobin was also positive in this case of inguinal metastases. Mammaglobin is reported to be the most reliable mammary-specific markers of which sensitivity and specificity are 84.3% and 85.0%, respectively [9]. Intriguingly, the series of CTs retrospectively reviewed showed the correlation of the growing patterns between the breast tumor and the inguinal lymph nodes (Fig. 2a, b). The dormancy of the swelling after the breast operation could be caused by the effect of the subsequent hormone therapy (Fig. 2c). Taking these immunohistochemical results as well as the course of progressing pattern of inguinal nodes into consideration, we diagnosed the present case as breast cancer origin. In our knowledge, this is the sixth reported case in English literature.

The first such report by Baba et al. [12] described a patient with inguinal node swelling preceding the ipsilateral breast lump by a year. She received intensive treatments including curative operations, chemotherapy, and radiation to the inguinal region. However, 32 months after the breast surgery, marked lymph node metastases recurred in the pelvis. She succumbed due to the entire organ metastases thereafter. The authors proposed two probable pathways to the inguinal lymph node metastasis: a direct pathway through skin or subcutaneous lymphatic vessels, or a retrograde pathway through submuscular fascia when axillary lymph nodes were blocked. Goyal et al. [15] also presented a case of right inguinal lymph node metastasis, 3 years after bilateral triple negative breast cancers. Local irradiation and taxane-based systemic chemotherapy were applied. Two years later, the developing extensive retroperitoneal and pelvic nodal metastases induced the patient to expire. The authors also speculated altered lymphatic pathways could have been responsible for the inguinal involvement.

There have been accumulating knowledges that the lymphatic pathways are relatively easy to be altered after axillary lymph node dissection and/or radiation [1–5]. Surprisingly, alteration of lymphatic pathways into the contralateral axilla [1, 2, 4, 5], paravertebral [4], or epigastric nodes [3] have been reported. Sato et al. [2] elucidated that either axillary dissection or radiation increased the rate of aberrant lymphatic drainage by examining the re-sentinel lymphoscintigraphy for the patients with ipsilateral breast cancer recurrences. Nine out of 17 patients (64.3%) receiving both axillary dissection and radiation showed the lymphatic drainage pattern into the contralateral axilla. Kaur et al. [1] examined 45 patients previously undergone breast-conserving surgery and complete ALND, who subsequently had reoperation for ipsilateral breast recurrence. Thirteen (29%) cases had a successful SLN biopsy, among which 5 cases were identified as showing non-axillary drainages: 3 in internal mammary and 2 in contralateral axillary nodes. Indeed, two out of 5 patients ended up being metastatic.

In the present case, however, the inguinal node metastases preceded the breast surgery. It is presumable that stagnated and elusive axillary lymphatics had already been existing preoperatively. The postoperative intractable lymphatic discharge shown in this case could be due to preexisting the stagnation of centrally heading lymphatic pathways. Additionally, the previously performed meticulous abdominal operations, her history of congenital hip location and extraordinary obesity could influence the changing lymphatic pathways. As there was no retroperitoneal or internal iliac lymph node metastasis, we speculate an alternate pathway through the body surface could be plausible. It probably passed from the subcutaneous or submuscular fascia to the contralateral epigastric lymphatics through the developed lymphatic networks by the previous five times’ abdominal operations and reached the superficial inguinal lymph nodes.

We acknowledge this is the first case of contralateral inguinal metastases from breast cancer reported in English literature. We need to mention that not only breast operation or radiation therapy, but abdominal surgery could cause alteration of the lymphatic flows of breast cancer. As shown in the previous cases, the prognoses of these patients were poor. Our patient has now been treated only by anti-estrogen therapy for adjuvant remedy due to her poor ADL and progressing dementia. Further continuous prudent follow-ups should be mandatory in the future.

## Conclusions

This case indicates that breast cancer can metastasize into inguinal lymph nodes, even to the contralateral side. The cause of the alterations of lymphatic pathways could be relevant to not only breast surgery, but also abdominal surgery. We need to consider the possibility of distant lymph node metastasis in the case of poly-treatments such as surgery and radiation for both chest and abdomen.

## Data Availability

Not applicable.

## Abbreviations

RT: Radiation therapy
ALND: Axillary lymph node dissection
CT: Computed tomography
PET: Positron emission tomography
FDG: Fluorodeoxyglucose
ADL: Activities of daily living
CK: Cytokeratin
SLN: Sentinel lymph node
ER: Estrogen receptor
PgR: Progesterone receptor
HER2: Human epidermal growth factor receptor 2
HE: Hematoxylin and eosin
